# Cerebrolysin and repetitive transcranial magnetic stimulation (rTMS) in patients with traumatic brain injury: a three-arm randomized trial

**DOI:** 10.3389/fnins.2023.1186751

**Published:** 2023-06-08

**Authors:** Olivia Verisezan Rosu, Nicoleta Jemna, Elian Hapca, Irina Benedek, Iulia Vadan, Ioana Muresanu, Diana Chira, Constantin Radu, Răzvan Cherecheş, Stefan Strilciuc, Dafin Muresanu

**Affiliations:** ^1^Department of Neurosciences, Iuliu Haţieganu University of Medicine and Pharmacy, Cluj-Napoca, Romania; ^2^RoNeuro Institute for Neurological Research and Diagnostic, Cluj-Napoca, Romania; ^3^Yale School of Public Health, Yale University, New Haven, CT, United States; ^4^Department of Public Health, Babeş-Bolyai University, Cluj-Napoca, Romania

**Keywords:** traumatic brain injury, Cerebrolysin, neurorecovery, neurotrophic factors, repetitive transcranial magnetic stimulation (rTMS)

## Abstract

**Introduction:**

Traumatic brain injury (TBI) is a major public health problem affecting millions worldwide. Despite significant advances in medical care, there are limited effective interventions for improving cognitive and functional outcomes in TBI patients.

**Methods:**

This randomized controlled trial investigated the safety and efficacy of combining repetitive transcranial magnetic stimulation (rTMS) and Cerebrolysin in improving cognitive and functional outcomes in TBI patients. Ninety-three patients with TBI were randomized to receive either Cerebrolysin and rTMS (CRB + rTMS), Cerebrolysin and sham stimulation (CRB + SHM), or placebo and sham stimulation (PLC + SHM). The primary outcome measures were the composite cognitive outcome scores at 3 and 6 months after TBI. Safety and tolerability were also assessed.

**Results:**

The study results demonstrated that the combined intervention of rTMS and Cerebrolysin was safe and well-tolerated by patients with TBI. Although no statistically significant differences were observed in the primary outcome measures, the descriptive trends in the study support existing literature on the efficacy and safety of rTMS and Cerebrolysin.

**Discussion:**

The findings of this study suggest that rTMS and Cerebrolysin may be effective interventions for improving cognitive and functional outcomes in TBI patients. However, limitations of the study, such as the small sample size and exclusion of specific patient populations, should be considered when interpreting the results. This study provides preliminary evidence for the safety and potential efficacy of combining rTMS and Cerebrolysin in improving cognitive and functional outcomes in TBI patients. The study highlights the importance of multidisciplinary approaches in TBI rehabilitation and the potential for combining neuropsychological measurements and interventions to optimize patient outcomes.

**Conclusion:**

Further research is needed to establish these findings’ generalizability and identify the optimal dosages and treatment protocols for rTMS and Cerebrolysin.

## Introduction

Traumatic brain injury (TBI) is a significant public health issue that affects millions of individuals worldwide, resulting in long-term disabilities that can affect thinking ability, emotional, behavioral, and personality disturbances, as well as other functional deficits (Coronado et al., 2015; Chan et al., 2022). Unfortunately, despite advancements in medical care, many individuals with TBI experience significant impairments in cognitive and physical functioning. These impairments can have a major impact on the individual’s quality of life and their ability to return to work and participate in social activities. Therefore, there is a pressing need for innovative interventions that can improve outcomes for these individuals (Maas et al., 2022).

We hypothesized that one potential approach to improve outcomes in traumatic brain injury (TBI) patients is combining Cerebrolysin treatment with repetitive transcranial magnetic stimulation (rTMS). Cerebrolysin is a peptide mixture derived from purified porcine brain proteins that has been shown to have neuroprotective and neurotrophic effects, as it may help protect and regenerate brain cells. After an acute brain lesion, there is always an endogenous continuous brain defense response consisting of two main sequences: an immediate one that aims to reduce brain damage, known as neuroprotection, and a later one that aims to repair the brain damage, known as neurorecovery. Neurotrophic factors are the most important endogenous molecules in brain protection and recovery. They modulate molecules with immediate pleiotropic neuroprotective activity and long-term multimodal effects (Muresanu et al., 2019). Due to this unique therapeutic effect, the principle of treatment with neurotrophic factors is based on repetitive periods of treatment in addition to acute administration. Cerebrolysin has a neurotrophic factor-like activity based on the four crucial endogenous neurobiological processes: neurotrophicity, neuroprotection, neuroplasticity, and neurogenesis. Additionally, this activity may have similar effects as the natural sequence of endogenous post-lesional regulation (Muresanu, 2009).

Repetitive transcranial magnetic stimulation (rTMS) is a non-invasive technique that uses magnetic fields to stimulate specific brain areas. Transcranial magnetic stimulation operates on Faraday’s principle of electromagnetic induction. Studies have shown the beneficial role of rTMS in neurorehabilitation, including motor recovery, spasticity reduction, depression treatment, and speech rehabilitation in TBI patients (Fitzgerald et al., 2009; Bonnì et al., 2014; Lüdemann-Podubecká et al., 2015; Chou et al., 2020). Although the effects of rTMS on cognition are based on the idea that stimulating different frequencies on a specific area can activate or deactivate specific regions or even networks and thus enhance or inhibit specific functions, experimental studies have also shown that rTMS can influence the molecular and cellular level, which can be independent of the induction of action potentials. One of the critical targets of rTMS for improving cognitive function was presumed to be the brain-derived neurotrophic factor (BDNF), as magnetic stimulation was thought to have a stimulating effect on its genetic expression. However, this hypothesis could not be confirmed when looking at serum BDNF levels after rTMS (Jiang and He, 2019).

While rTMS has been extensively studied for cognitive rehabilitation in other diseases, there is limited and mixed research on its efficacy for cognitive rehabilitation in TBI (Pink et al., 2021). The main area of concern regarding the use of TMS in stroke or TBI patients has been the triggering of kindling activity, which can induce seizures. Seizure induction, however, has rarely been reported following rTMS, and animal studies have shown no clear evidence that rTMS leads to increased seizure susceptibility (Miniussi and Rossini, 2011). While cognitive treatment with repetitive transcranial magnetic stimulation (rTMS) has been tested in Alzheimer’s disease, the combination of rTMS and cognitive treatment appears to benefit cognition (Bentwich et al., 2011). However, limited data is available regarding the efficacy of rTMS as an add-on to pharmacological treatment in cognitive rehabilitation (Haffen et al., 2012; Maas et al., 2022). Studies on rTMS as an add-on treatment for depression had shown promising results when high-frequency magnetic stimulation was used. However, further research is needed to determine the optimal frequency, intensity, and duration of rTMS treatment to improve cognitive outcomes in TBI rehabilitation (Wei et al., 2017).

This trial evaluated the safety and efficacy of combining Cerebrolysin treatment with repetitive transcranial magnetic stimulation (rTMS) for patients with traumatic brain injury (TBI). This study represented the first investigation into the effectiveness of combined rTMS and pharmacological intervention (CRB) for cognitive rehabilitation in TBI. Its multi-arm design was aimed to identify which intervention or combination of interventions is most effective while also allowing for a comparison of the individual effects of each intervention. The primary objective of this clinical study was to explore the feasibility, efficacy, and safety of combining rTMS and Cerebrolysin treatment versus CRB alone on a battery of co-primary neurocognitive outcomes at 3 and 6 months after TBI.

## Materials and methods

This study was a phase II, single-center, randomized, double-blind, placebo-controlled study conducted as part of doctoral studies in Cluj-Napoca, Romania. The study protocol was approved by the Ethics Committee of the Iuliu Hatieganu University of Medicine and Pharmacy (8 Babeş Street, 400012 Cluj-Napoca, Romania; +40-264-597-256; contact@umfcluj.ro) on 01/02/2018, reference no. 2/08.01.2018. The protocol was amended on 171/02.04.2018 (ref. no. 133/15.03.2018) and on 30/05/2019 (ref. no. 118/23.04.2019), extending the inclusion criteria and study end date to facilitate the inclusion of patients. The trial followed the Declaration of Helsinki and Good Clinical Practice guidelines. All participants provided written informed consent before enrollment. The protocol and subsequent trial information may be found in the ISRCTN clinical trial registry (ISRCTN, n.d.). The manuscript is reported in line with the extended version of the CONSORT 2010 statement on reporting of multi-arm parallel-group randomized trials (Juszczak et al., 2019).

Participants were recruited between April 2018 and September 2021 from patients with moderate-severe TBI aged between 18 and 70 years who had an onset of TBI within 30 days before screening. All patients with TBI were initially admitted to hospital, they were evaluated by clinicians and followed standard treatment. Study visits and study treatment administration lasted 180 days and were conducted in an outpatient setting at the RoNeuro Institute for Neurological Research and Diagnostic, Cluj-Napoca, Romania. Participants were excluded if they had any contraindication to rTMS or Cerebrolysin treatment. The trial inclusion criteria consisted of patients with traumatic brain injury onset within 30 days before screening, CT/MRI-confirmed focal or diffuse lesions, age 18–70 years (updated 28/02/2020: 18–80 years), Pre-Trauma Karnofsky Index 100, and willingness to comply with the protocol requirements for the duration of the study. Patients with metal implants in the head or within the stimulation area, medical implanted devices (cardiac pacemaker, cochlea implant or medication pumps), history of intracranial interventions, evidence of pre-existing major health problems (e.g., cancer, hematological, renal, hepatic, or coronary disease, psychiatric disorder, diabetes, myocardial infarction or other known heart diseases, disabling or musculoskeletal problems like rheumatoid arthritis, epilepsy, evidence of degenerative or inflammatory diseases affecting nervous system), history of intracranial interventions, any neurological or non-neurological condition independent from TBI that might influence the functional outcome or other efficacy outcome measures, injury of writing hand influencing cognitive or other outcome measures, clear clinical signs of intoxication influencing the evaluation, major drug dependency including alcohol, chronic treatment with steroids, Ca2 + -channel blockers or major anticoagulants (e.g., warfarin and other coumarin derivates), monoamine oxidase inhibitors, antipsychotic drugs or nootropic molecules, patients with penetrating brain injury, females who are pregnant or lactating, or females who are of childbearing potential and not taking adequate contraceptive precautions were excluded from the study. Females of childbearing potential taking acceptable contraceptive precautions were included in the study.

The study schedule consisted of three visits for each participant: Screening and Baseline at Study Day 30, Visit 1 at Study Day 101, and Visit 2 at Study Day 180. There was no follow-up conducted after the 180-day evaluation. Patients who met the inclusion and exclusion criteria were assigned a random number based on a pre-generated list by a third-party service (blocks of three, equal allocation to groups). Sealed and opaque randomization envelopes were provided to the study center based on the random list, the person responsible for preparing the ready-to-use infusion, the person administering the rTMS protocol, and the study coordinator. Participants were randomly allocated to one of three groups: (1) Cerebrolysin and rTMS–CRB + rTMS, (2) Cerebrolysin and sham rTMS - CRB + SHM, or (3) placebo and sham rTMS–PLC + SHM. All groups received standard care after traumatic brain injury. All participants received three cycles of treatment. The first group received 30 ml of Cerebrolysin infusions + rTMS for 10 days at Days 31–40, 61–70, and 91–100. The second group received 30 ml of Cerebrolysin infusions + sham rTMS for the same duration and days as the first group, while the third group received a placebo of 250 ml of 0.9% saline solution + sham rTMS for the same duration and days as the other groups. The Cerebrolysin was diluted in 0.9% saline solution up to 250 ml, and the rTMS stimulation parameters for left dorsolateral prefrontal cortex (DLPFC) were 10 Hz (10 stimuli/second), 1,200 stimuli/day, with a total session time of 33 min–40 trains of 3 sec interleaved by a pause of 20 s, and an intensity of 120% of resting motor threshold. The resting motor threshold was determined at the beginning of the first treatment session and was defined as the minimal intensity at which at least five of 10 motor-evoked potentials were 50 μV in amplitude in the pollicis abductor brevis. Repetitive stimulation was delivered using the medical device MagPro X100 (MagVenture, Farum, Denmark) with a figure-8 coil (MCF-B65), held tangential to the scalp with the handle pointing upward. The DLPFC was localized using the 10–20 EEG system, with the coil at F3. Sham stimulation was performed with a sham-coil (MCF-P-B 65, MagVenture, Farum, Denmark) with a mechanical outline and sound level identical to MCF-B65, providing the same cutaneous discomfort and muscle twitching level as real stimulation. Two rTMS technicians administered both sham and real rTMS without being involved in any other study-related procedures or allowed to disclose information about the treatment procedure.

The primary outcome measures consisted of 10 cognitive function tests, which were assessed by a team of neuropsychologists using the Stroop Color-Word Test (Scarpina and Tagini, 2017), Montreal Cognitive Assessment (MoCA) (Hobson, 2015), two subscales of the Wechsler adult intelligence scale (3rd edition): Processing Speed Index (PSI) and Digit Span (Wechsler, n.d.), Trail Making Test (Bucks, 2013), three subscales of the Cambridge Neuropsychological Test Automated Battery (CANTAB): One Touch Stockings of Cambridge, The Multitasking Test, and Reaction Time (Sandberg, 2011), the Hamilton Anxiety Rating Scale (HARS) (Hamilton, 1959) and the Hamilton Rating Scale for Depression (HDRS) (Hamilton, 1960). These tests were conducted on days 30, 101, and 180 of the study. Adverse events of the interventions were recorded using a safety report form based on patient self-reports during the entire duration of the trial. Secondary outcomes consisted of eye movements assessed using a Tobii Pro TX300 eye-tracking device and brain electrical activity assessed using electroencephalography (EEG). Since these secondary measures require distinct data processing and specialized analysis, they will be reported in subsequent manuscripts.

The trial was conducted under double-blind conditions to ensure the blinding of investigators, study personnel, and patients to treatment allocation. To maintain blinding, colored infusion lines were used for drug administration as Cerebrolysin is an amber-colored solution. Patients who met the inclusion and exclusion criteria were assigned a random number corresponding to the pre-generated random list prepared by a biometrician selected by the sponsor. Each patient received a set of envelopes for their treatment, which was distributed to the study nurse responsible for preparing the infusion solution. The study nurse was only responsible for the preparation and administration of infusion solutions and was not involved in any other study-related procedures or allowed to disclose information about treatment allocation. The treatment envelope was not opened until the patient’s first ready-to-use infusion was prepared. The database was closed, and analysis populations were determined before unblinding the whole study.

The study’s power was determined based on certain specifications. These included a one-sided type I error of alpha = 0.05, a type II error of β = 0.2, and a medium-sized effect size according to Cohen. The estimated correlations among the outcome scales were ρ = 0.4. Sample size calculations were performed using non-parametric methods with the Nnpar 1.0 software from idv Data Analysis and Study Planning. The total required sample size for the study was determined to be 30 patients per group, including a 10% enhancement for potential dropouts. This sample size allows for the detection of a medium-sized difference between groups with an 80% power. Due to the exploratory nature of this trial, aimed to test the feasibility and safety of the concomitant intervention, interim power calculations or extensions of the trials were not performed.

Before unblinding the study, a blind review was conducted, in which protocol violations were classified as “severe,” “major,” “minor,” or “none,” and patients were allocated to individual data sets based on the classification of possible protocol violations. The analysis populations included safety, intention-to-treat (ITT), and per protocol (PP). The safety population included all patients who received at least one dose of study medication and had at least one contact with the investigator afterward. The ITT population was defined as all patients who had no “severe” violation of entry criteria, received at least one dose of medication, and had at least one post-baseline observation of at least one primary efficacy criterion (“modified” ITT). The PP population included all patients who were eligible for ITT evaluation and did not show major protocol deviations. A sensitivity analysis was performed for the PP data set as an exploratory approach. Homogeneity analyses for baseline were performed based on the ITT population. In case of heterogeneities, stratified analyses were performed as second-line analyses. Patients with compliance for the entire study below 80% for the treatments were considered protocol violators and were not included in the per protocol analysis. Based on good clinical practice recommendations on the conduct of clinical trials during the COVID-19 pandemic, the study team decided to delay study visits rather that terminate the study. Visit delays during the 2020 lockdown (*n* = 16; 15 for Visit 2, 1 for Visit 3) were included in the PP analysis, provided they did not have other violations. Due to the low sample size, any sensitivity analysis excluding these cases would not bring additional information.

Statistical procedures were performed using the R programming language and SAS^®^ On Demand for Academics (Ogle et al., n.d.). Descriptive statistics and graphs were generated for the ITT population. We performed the Kruskal-Wallis Rank Sum Test for both ITT and PP populations to assess the statistical significance of the differences across groups. The pairwise comparisons were performed using Dunn’s test for multiple comparisons with Bonferroni adjustment. Safety analyses were conducted on the ITT population and included the incidence of adverse events and serious adverse events.

The primary analysis compared the three groups’ baseline changes in composite neurocognitive outcomes. All variables were included in the Kruskal-Wallis analysis. For each patient, we calculated the differences between Visit 1 (Day 101) and baseline and Visit 2 (Day 180) and baseline across all variables and used z-scores to create a composite cognitive total score (Andrade, 2021).

## Results

The study enrolled 93 individuals who had suffered traumatic brain injury 30 days before screening. The ITT population, used for all efficacy analyses, included patients with at least one dose of medication and at least one post-baseline observation of at least one primary efficacy criterion. The PP population was defined as all patients eligible for ITT evaluation and who did not show significant protocol deviations. Violation of inclusion or exclusion criteria was not observed in the sample. Premature discontinuations were reported in 9 patients due to loss to follow-up (9.6%). The ITT population is comprised of 86 TBI patients.

**FIGURE 1:**
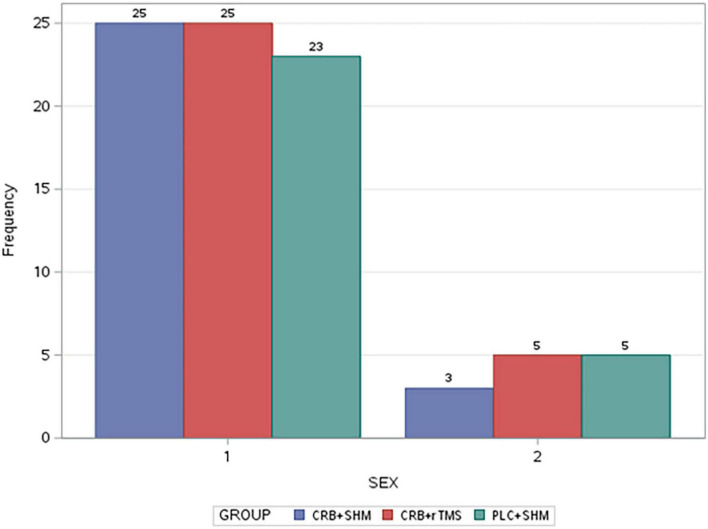
Gender distribution of study groups (2-female; 1-male). CRB, Cerebrolysin; SHM, sham repetitive transcranial magnetic stimulation; rTMS, repetitive transcranial magnetic stimulation; PLC, placebo.

In contrast, the PP population comprises 84 patients (loss to follow-up from ITT–CRB + SHM: 1, PLC + SHM: 1). The safety population included all patients with at least one dose of study medication and one subsequent contact with study investigators (86 patients). Study populations were comparable in gender distribution, which was unbalanced at a 4:1 male-to-female ratio (Figure 1). The composite outcome analysis is based on the ITT set without missing data (*n* = 64).

**FIGURE 2:**
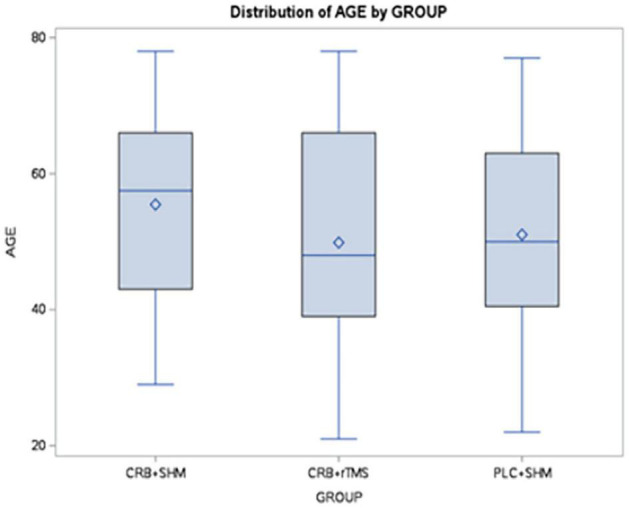
Distribution of age distribution across study groups using boxplots (boxes represent the middle 50% of the data; horizontal lines–means; squares–medians; whiskers–1.5× interquartile range from the upper and lower quartiles). CRB, Cerebrolysin; SHM, sham repetitive transcranial magnetic stimulation; rTMS, repetitive transcranial magnetic stimulation; PLC, placebo.

The group with the highest mean age was CRB + SHM (mean = 55.46 years), followed by PLC + SHM (mean = 51.04 years), and CRB + rTMS (mean = 49.87 years). Boxplots of age distribution across study groups (Figure 2).

In total, 21 adverse events (AE) were reported during this trial for 7 patients in the CRB + SHM group, 6 in the CRB + rTMS group, and 8 in the PLC + SHM group. Among adverse events, we remarked symptoms related to the traumatic brain injury itself or other associated health conditions, Covid infections, and other types of infections. Also, for 4 patients there were reported certain symptoms, like headache, dizziness, or fatigue, already associated in the literature with Cerebrolysin administration or rTMS. Nevertheless, they were described as mild symptoms, they did not require concomitant treatment or interruption of study medication, and the outcome was favorable. One serious adverse event (SAE) was reported for the CRB + SHM group. Based on the assessment of study investigators, the SAE was not related to the administration of the intervention. Non-parametric group differences were not statistically significant (Kruskal–Wallis chi-squared = 0.57734, df = 2, *p*-value = 0.7493 for AE, and Kruskal–Wallis chi-squared = 2.0714, df = 2, *p*-value = 0.355 for SAE). Results from *post-hoc* Dunn’s test for multiple comparisons for both AE and SAE are available in Table 1.

**TABLE 1:** Multiple pairwise comparisons between study group AEs and SAEs using Dunn’s test.

	AE			SAE		
**Comparison**	***Z***	**P.unadj**	**P.adj**	***Z***	**P.unadj**	**P.adj**
CRB + rTMS–CRB + SHM	−0.4403399	0.6596910	1	−1.260425	0.2075163	0.6225488
CRB + rTMS–PLC + SHM	−0.7548684	0.4503280	1	0.000000	1.0000000	1.0000000
CRB + SHM–PLC + SHM	−0.3092419	0.7571375	1	1.239239	0.2152569	0.6457706

**FIGURE 3:**
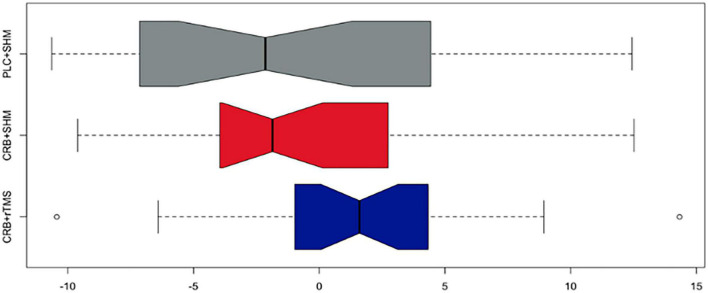
Baseline difference composite cognitive outcome (z-scores) in the ITT population after the second visit on study day 101 across study groups (colors represent study groups; boxes represent the middle 50% of the data; horizontal lines–means; whiskers–1.5× interquartile range from the upper and lower quartiles; notches represent 95% confidence intervals; 0 = sample mean z-score). CRB, Cerebrolysin; SHM, sham repetitive transcranial magnetic stimulation; rTMS, repetitive transcranial magnetic stimulation; PLC, placebo.

**FIGURE 4:**
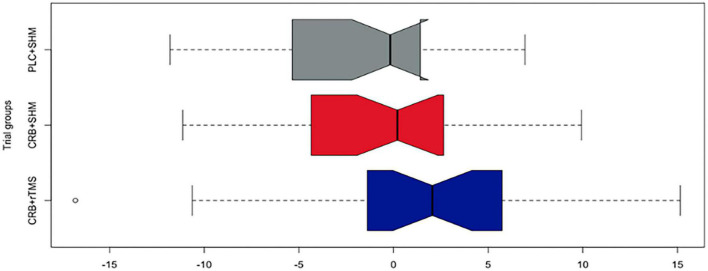
Baseline difference composite cognitive outcome (z-scores) in the ITT population after the second visit on study day 180 across study groups (colors represent study groups; boxes represent the middle 50% of the data; horizontal lines–means; whiskers–1.5× interquartile range from the upper and lower quartiles; notches represent 95% confidence intervals; 0 = sample mean z-score). CRB, Cerebrolysin; SHM, sham repetitive transcranial magnetic stimulation; rTMS, repetitive transcranial magnetic stimulation; PLC, placebo.

Composite outcomes in the ITT population for Visit 2 and Visit 3 were compiled based on z-scores of each outcome scale baseline differences (*n* = 64: PLC + SHM: 24, CRB + SHM:20, CRB + rTMS:20, less than the total sample size due to missing data). The Kruskal–Wallis test conducted to compare the scores of three groups on the composite cognitive outcome revealed no significant differences among the groups (Day 101–χ^2^ = 5.732, *p* = 0.06; Day 180 χ^2^ = 5.833, *p* = 0.54). The three groups included participants who received CRB + rTMS, CRB + SHM, and PLC + SHM interventions. Results from *post-hoc* Dunn’s test for multiple comparisons for both Day 101 and Day 180 baseline differences in composite cognitive outcome are available in Table 2. Despite descriptive superiority for both intervention groups, non-parametric tests were not statistically significant for the combined outcome (Figures 3, 4).

**TABLE 2:** Multiple pairwise comparisons between study group composite cognitive outcome (z-scores) using Dunn’s test.

	Day 101			Day 180		
**Comparison**	***Z***	**P.unadj**	**P.adj**	***Z***	**P.unadj**	**P.adj**
CRB + rTMS–CRB + SHM	1.6688222	0.09515263	0.28545788	1.8430467	0.06532219	0.19596658
CRB + rTMS–PLC + SHM	2.3373784	0.01941951	0.05825854	2.2295095	0.02578002	0.07734006
CRB + SHM–PLC + SHM	0.6413424	0.52130024	1.00000000	0.3799672	0.70396976	1.00000000

Individual outcomes scales are similar to composite findings. More information is available in the Supplementary Annex 1. Given the low sample size of the trial that would inherently lead to the low power of reported analyses, no further hypothesis testing was performed.

## Discussion

The primary aim of this clinical study was to investigate the practicability, effectiveness, and safety of administering rTMS and Cerebrolysin in combination compared to CRB alone. In addition, the study evaluated several co-primary neurocognitive outcomes at 3 and 6 months following TBI. Despite the challenges posed by the COVID-19 pandemic, this clinical study was successfully conducted, and data were collected from all participants. The study team implemented various measures, such as antigen screening and specialized protective equipment, to ensure the safety of participants and staff while maintaining the integrity of the study. However, there were delays in some of the in-person visits, which may have impacted the accuracy of the data collected by introducing a disadvantage for active treatment arms as the window for neurorecovery decreases following brain injury (Bernhardt et al., 2017). While these limitations were acknowledged in the manuscript, it is also essential to acknowledge the challenges faced by clinical research during the pandemic.

Our findings highlight that the combination of rTMS and Cerebrolysin is safe and well tolerated by patients with traumatic brain injury. While primary outcomes and their subscales did not show statistically significant differences, the descriptive trends in this study support existing literature on the efficacy and safety of both rTMS and Cerebrolysin (Muresanu et al., 2016; Guekht et al., 2017; Beghi et al., 2021; Pink et al., 2021; Mureşanu D. F. et al., 2022; Mureşanu I. A. et al., 2022).

The hypothesis for this study was based on findings of the CAPTAIN trial series (Poon et al., 2019; Muresanu et al., 2020; Vester et al., 2021), which showed a significant effect of Cerebrolysin on a multidimensional ensemble of outcomes in patients who had suffered moderate-severe TBI. While study populations in our trial are similar to the CAPTAIN project, the model of treatment administration is fundamentally different, as interventions are initiated 30 days after injury (i.e., chronic, intermittent admission), as opposed to acute initiation (i.e., 4–6 h after hospital admission). This allowed us to provide insight into the ability of the interventions to operate in patients in the chronic stage of illness. The second important difference between the two projects is the control for TBI severity in the emergency setting. As patients enrolled in our study were included in a third-party outpatient clinic 1 month after acute presentation, information such as admission Glasgow Coma Scale scores (i.e., a key clinical prognostic factor for TBI outcome and neurorehabilitation potential) or other data gathered from the emergency setting were not available. Hence, in contrast with the CAPTAIN trial, which had Baseline Prognostic Risk Score (BPRS) as a tool for baseline comparability, our sample has an unknown distribution of TBI severity, which could have confounded results similar to the age imbalance present between study groups (i.e., the Cerebrolysin and sham group was roughly five years older, on average).

While paradigm-shifting clinical implications based solely on this study cannot be drawn, findings suggest that future studies may show the potential to improve cognitive and motor function in patients with TBI. Based on our observations, limited potential risks are associated with the combined intervention after TBI. These findings align with existing literature (Bornstein et al., 2018; Kim and Paik, 2020; Kletzel et al., 2020; Strilciuc et al., 2021). Therefore, both Cerebrolysin and its neuromodulatory enhancement by rTMS should be considered as candidates when deciding a neurorehabilitation strategy for TBI in patients who have access to such interventions. These should also be discussed in clinical guidelines.

Our study also provides valuable epistemological insight into clinical trial methodology in neurotrauma. While lessons from the CAPTAIN trial, such as the importance of multidimensional outcome assessment, have been incorporated into our design, the absence of stratification based on acute severity remains an important feature that should not be omitted in similar work in the future. In addition, other limitations may have further influenced our results, which should also be considered. For example, the sample size was relatively small, and the study was conducted at a single site, which may limit the generalizability of the findings to larger populations. Additionally, our study’s duration was relatively short, and longer-term follow-up studies are necessary to determine the treatment’s long-term efficacy. Additionally, the exclusion of specific patient populations, such as those with more mild TBI or pre-existing medical conditions, may limit the applicability of the findings to these groups. Considering these limitations when interpreting the study’s results and applying them to clinical practice is essential. The study’s strengths include its rigorous design and adherence to Good Clinical Practice guidelines. The blinding procedures minimized the potential for bias in the study’s findings. Using both rTMS and Cerebrolysin allowed for a comprehensive examination of these treatments’ potential benefits and risks in TBI patients.

However, much remains to be learned about the optimal combined use of rTMS and Cerebrolysin. Overall, there is a great need for continued research in this area to understand better the optimal use of such interventions in TBI patients and further improve outcomes for this vulnerable patient population. Multi-center studies with larger sample sizes are needed to confirm the efficacy of rTMS and Cerebrolysin in TBI patients. While the current study provides promising results, more extensive studies with more diverse patient populations are needed to establish the generalizability of these findings. In addition to larger, confirmatory trials that could identify the effect size for the intervention, further studies could explore the optimal dosages and treatment protocols for rTMS and Cerebrolysin, as well as their long-term effects on patients with TBI.

Moreover, future studies could explore potential subgroup effects, such as the effects of rTMS and Cerebrolysin in patients with varying degrees of injury severity or different ages. This information could help to identify which patients are most likely to benefit from these interventions and tailor treatment accordingly. Given that TBI is a complex disorder with a range of cognitive and functional deficits, future research could explore the potential benefits of combining rTMS and Cerebrolysin with other interventions, such as cognitive rehabilitation or pharmacotherapy. This could help maximize these interventions’ potential benefits and improve outcomes for TBI patients.

In conclusion, our study provides preliminary evidence for the safety and potential efficacy of combining rTMS and Cerebrolysin in improving cognitive and functional outcomes in TBI patients. This combined intervention may synergistically promote neuroplasticity and neural repair in TBI patients. However, further research is needed to confirm these findings and explore potential subgroup effects. The study’s limitations, such as the small sample size and exclusion of specific patient populations, should also be considered when interpreting the results. Nonetheless, the study’s rigorous design and adherence to Good Clinical Practice guidelines provide confidence in the validity of the findings. This study also highlights the importance of multidisciplinary approaches in TBI rehabilitation and the potential for combining neuropsychological measurements and interventions to optimize patient outcomes.

## Data availability statement

Replication data for this analysis is published in the Harvard Dataverse, accessible at https://doi.org/10.7910/DVN/DOFCIV.

## Ethics statement

The studies involving human participants were reviewed and approved by the Ethics Committee of the Iuliu Haţieganu University of Medicine and Pharmacy. The patients/participants provided their written informed consent to participate in this study.

## Author contributions

DM, SS, and OV conceived and designed the analysis. OV, NJ, IV, IM, IB, and EH collected the data. DM and SS contributed data or analysis tools. SS, DC, RC, and CR performed the analysis. OV, SS, and DC wrote the manuscript. All authors read and approved the manuscript.
