# Supplementary material for: Cerebrolysin and repetitive transcranial magnetic stimulation (rTMS) in patients with traumatic brain injury: a three-arm randomized trial
Source: Front Neurosci. 2023 Jun 8;17:1186751. doi: 10.3389/fnins.2023.1186751 (PMC10285097; doi:10.3389/fnins.2023.1186751)
Supplement: Supplementary file 1 [file Data_Sheet_1.PDF]

Annex 1: Group differences

	Intention to treat (ITT) - N=86	Per protocol (PP) - N=84
**MOCA_v1-baseline**	Kruskal-Wallis chi-squared = 2.9946, df = 2, p-value = 0.2237<br>Gr. comp. Adj. p-value<br>1-2 0.3051591<br>1-3 0.5989947<br>2-3 1.0000000	Kruskal-Wallis chi-squared = 1.1016, df = 2, p-value = 0.5765<br>Gr. comp. Adj. p-value<br>1-2 0.8862243<br>1-3 1.0000000<br>2-3 1.0000000
**MOCA_v2-baseline**	Kruskal-Wallis chi-squared = 2.954, df = 2, p-value = 0.2283<br>Gr. comp. Adj. p-value<br>1-2 0.7156052<br>1-3 0.2869016<br>2-3 1.0000000	Kruskal-Wallis chi-squared = 1.5472, df = 2, p-value = 0.4614<br>Gr. comp. Adj. p-value<br>1-2 0.6790492<br>1-3 1.0000000<br>2-3 1.0000000
**PSI_DSC_v1-baseline**	Kruskal-Wallis chi-squared = 1.5716, df = 2, p-value = 0.4557<br>Gr. comp. Adj. p-value<br>1-2 0.7682078<br>1-3 1.0000000<br>2-3 0.9025362	Kruskal-Wallis chi-squared = 1.5482, df = 2, p-value = 0.4611<br>Gr. comp. Adj. p-value<br>1-2 0.8645722<br>1-3 0.8295927<br>2-3 1.0000000
**PSI_DSC_v2-baseline**	Kruskal-Wallis chi-squared = 0.17898, df = 2, p-value = 0.9144<br>Gr. comp. Adj. p-value<br>1-2 1.0000000<br>1-3 1.0000000<br>2-3 1.0000000	Kruskal-Wallis chi-squared = 2.9083, df = 2, p-value = 0.2336<br>Gr. comp. Adj. p-value<br>1-2 0.9917768<br>1-3 0.2690619<br>2-3 1.0000000
**PSI_SS_v1-baseline**	Kruskal-Wallis chi-squared = 5.7111, df = 2, p-value = 0.05752<br>Gr. comp. Adj. p-value<br>1-2 0.0507960<br>1-3 0.8456964<br>2-3 0.5910859	Kruskal-Wallis chi-squared = 3.5939, df = 2, p-value = 0.1658<br>Gr. comp. Adj. p-value<br>1-2 0.1821421<br>1-3 1.0000000<br>2-3 0.6930949
**PSI_SS_v2-baseline**	Kruskal-Wallis chi-squared = 3.4516, df = 2, p-value = 0.178<br>Gr. comp. Adj. p-value<br>1-2 0.1898350<br>1-3 1.0000000<br>2-3 0.9600965	Kruskal-Wallis chi-squared = 1.4536, df = 2, p-value = 0.4835<br>Gr. comp. Adj. p-value<br>1-2 0.8495979<br>1-3 1.0000000<br>2-3 0.9061589

**SCWT_W-D_v1-baseline**	Kruskal-Wallis chi-squared = 0.43139, df = 2, p-value = 0.806<br>Gr. comp. Adj. p-value<br>1-2 1.0000000<br>1-3 1.0000000<br>2-3 1.0000000	Kruskal-Wallis chi-squared = 0.76864, df = 2, p-value = 0.6809<br>Gr. comp. Adj. p-value<br>1-2 1.0000000<br>1-3 1.0000000<br>2-3 1.0000000
**SCWT_W-D_v2-baseline**	Kruskal-Wallis chi-squared = 0.19297, df = 2, p-value = 0.908<br>Gr. comp. Adj. p-value<br>1-2 1.0000000<br>1-3 1.0000000<br>2-3 1.0000000	Kruskal-Wallis chi-squared = 1.3469, df = 2, p-value = 0.5099<br>Gr. comp. Adj. p-value<br>1-2 1.0000000<br>1-3 1.0000000<br>2-3 0.8497795

**SCWT_C-D_v1-baseline**	Kruskal-Wallis chi-squared = 1.0735, df = 2, p-value = 0.5846<br>Gr. comp. Adj. p-value<br>1-2 1.0000000<br>1-3 1.0000000<br>2-3 1.0000000	Kruskal-Wallis chi-squared = 1.9222, df = 2, p-value = 0.3825<br>Gr. comp. Adj. p-value<br>1-2 1.0000000<br>1-3 0.4968267<br>2-3 1.0000000
**SCWT_C-D_v2-baseline**	Kruskal-Wallis chi-squared = 1.8311, df = 2, p-value = 0.4003<br>Gr. comp. Adj. p-value<br>1-2 0.9214534<br>1-3 0.6094999<br>2-3 1.0000000	Kruskal-Wallis chi-squared = 1.2103, df = 2, p-value = 0.546<br>Gr. comp. Adj. p-value<br>1-2 0.9124396<br>1-3 1.0000000<br>2-3 1.0000000
**DS_F_v1-baseline**	Kruskal-Wallis chi-squared = 0.77618, df = 2, p-value = 0.6784<br>Gr. comp. Adj. p-value<br>1-2 1.0000000<br>1-3 1.0000000<br>2-3 1.0000000	Kruskal-Wallis chi-squared = 3.4141, df = 2, p-value = 0.1814<br>Gr. comp. Adj. p-value<br>1-2 1.0000000<br>1-3 0.2565390<br>2-3 0.4519643
**DS_F_v2-baseline**	Kruskal-Wallis chi-squared = 4.4426, df = 2, p-value = 0.1085<br>Gr. comp. Adj. p-value<br>1-2 0.1927498<br>1-3 0.2241638<br>2-3 1.0000000	Kruskal-Wallis chi-squared = 3.4781, df = 2, p-value = 0.1757<br>Gr. comp. Adj. p-value<br>1-2 1.0000000<br>1-3 0.1956700<br>2-3 0.7596824
**DS_B_v1-baseline**	Kruskal-Wallis chi-squared = 3.368, df = 2, p-value = 0.1856<br>Gr. comp. Adj. p-value<br>1-2 1.0000000<br>1-3 0.2004813<br>2-3 1.0000000	Kruskal-Wallis chi-squared = 1.1998, df = 2, p-value = 0.5489<br>Gr. comp. Adj. p-value<br>1-2 1.0000000<br>1-3 1.0000000<br>2-3 1.0000000
**DS_B_v2-baseline**	Kruskal-Wallis chi-squared = 7.2622, df = 2, p-value = 0.02649<br>Gr. comp. Adj. p-value<br>1-2 0.3176149<br>1-3 0.0227401<br>2-3 0.9236916	Kruskal-Wallis chi-squared = 6.082, df = 2, p-value = 0.04779<br>Gr. comp. Adj. p-value<br>1-2 0.4821642<br>1-3 0.0423081<br>2-3 0.9716367
**TMT1_v1-baseline**	Kruskal-Wallis chi-squared = 1.979, df = 2, p-value = 0.3718<br>Gr. comp. Adj. p-value<br>1-2 0.5117596<br>1-3 1.0000000<br>2-3 1.0000000	Kruskal-Wallis chi-squared = 2.6055, df = 2, p-value = 0.2718<br>Gr. comp. Adj. p-value<br>1-2 0.6528185<br>1-3 0.3894484<br>2-3 1.0000000

**TMT1_v2-baseline**	Kruskal-Wallis chi-squared = 6.4319, df = 2, p-value = 0.04012<br>Gr. comp. Adj. p-value<br>1-2 0.1267959<br>1-3 0.0617833<br>2-3 1.0000000	Kruskal-Wallis chi-squared = 2.782, df = 2, p-value = 0.2488 Gr. comp. Adj. p-value<br>1-2 0.3125658<br>1-3 0.7885215<br>2-3 1.0000000

**TMT2_v1-baseline**	Kruskal-Wallis chi-squared = 0.26422, df = 2, p-value = 0.8762<br>Gr. comp. Adj. p-value<br>1-2 1.0000000<br>1-3 1.0000000<br>2-3 1.0000000	Kruskal-Wallis chi-squared = 1.4481,df = 2, p-value = 0.4848<br>Gr. comp. Adj. p-value<br>1-2 1.0000000<br>1-3 1.0000000<br>2-3 0.7546505
**TMT2_v2-baseline**	Kruskal-Wallis chi-squared = 2.469, df = 2, p-value = 0.291<br>Gr. comp. Adj. p-value<br>1-2 0.8008163<br>1-3 1.0000000<br>2-3 0.3823402	Kruskal-Wallis chi-squared = 2.8738,df = 2, p-value = 0.2377<br>Gr. comp. Adj. p-value<br>1-2 0.5692837<br>1-3 1.0000000<br>2-3 0.3263420
**HDRS_v1-baseline**	Kruskal-Wallis chi-squared = 3.1861, df = 2, p-value = 0.2033<br>Gr. comp. Adj. p-value<br>1-2 1.0000000<br>1-3 0.4099455<br>2-3 0.3252967	Kruskal-Wallis chi-squared = 2.2428,df = 2, p-value = 0.3258<br>Gr. comp. Adj. p-value<br>1-2 1.0000000<br>1-3 0.4993261<br>2-3 0.7122785
**HDRS_v2-baseline**	Kruskal-Wallis chi-squared = 2.4418, df = 2, p-value = 0.295<br>Gr. comp. Adj. p-value<br>1-2 1.0000000<br>1-3 0.9265261<br>2-3 0.3738440	Kruskal-Wallis chi-squared = 1.1329,df = 2, p-value = 0.5675<br>Gr. comp. Adj. p-value<br>1-2 1.0000000<br>1-3 1.0000000<br>2-3 0.9894535
**HADS_v1-baseline**	Kruskal-Wallis chi-squared = 1.0283, df = 2, p-value = 0.598<br>Gr. comp. Adj. p-value<br>1-2 1.0000000<br>1-3 0.9529634<br>2-3 1.0000000	Kruskal-Wallis chi-squared = 1.31, df =2, p-value = 0.5194<br>Gr. comp. Adj. p-value<br>1-2 1.0000000<br>1-3 0.7652385<br>2-3 1.0000000
**HADS_v2-baseline**	Kruskal-Wallis chi-squared = 0.42942, df = 2, p-value = 0.8068<br>Gr. comp. Adj. p-value<br>1-2 1.0000000<br>1-3 1.0000000<br>2-3 1.0000000	Kruskal-Wallis chi-squared = 1.1456,df = 2, p-value = 0.5639<br>Gr. comp. Adj. p-value<br>1-2 1.0000000<br>1-3 1.0000000<br>2-3 1.0000000

Group Reference:

- 1 = Cerebrolysin + rTMS
- 2 = Cerebrolysin + sham
- 3 = Placebo + sham
